# Antioxidant and protective effects of Royal jelly on histopathological changes in testis of diabetic rats

**Published:** 2016-08

**Authors:** Elham Ghanbari, Vahid Nejati, Mozafar Khazaei

**Affiliations:** 1 *Fertility and Infertility Research Center, Kermanshah University of Medical Sciences, Kermanshah, Iran.*; 2 *Department of Biology, Faculty of Science, Urmia University, Urmia, Iran. *

**Keywords:** *Diabetes mellitus*, *Royal jelly*, *Streptozotocin*, *Testis*, *Histopathology*

## Abstract

**Background:**

Diabetes is the most common endocrine disease. It has adverse effects on male reproductive function. Royal Jelly (RJ) has antioxidant and anti-diabetic effects and show protective effects against diabetes.

**Objective:**

This study was conducted to evaluate the effect of RJ on histopathological alterations of the testicular tissue in streptozotocin (STZ)-induced diabetic rats.

**Materials and Methods:**

In this experimental study, 28 adult Wistar rats were randomly divided into control (C), royal jelly (R), diabetic (D) and RJ-treated diabetic (D+R) groups. Diabetes was induced by a single intraperitoneal injection of STZ at 50 mg/kg body weight (BW). The rats from the R and D+R groups received daily RJ (100 mg/kg BW) for 6 wks orally. Hematoxylin-Eosin staining was used to analyze histopathological changes including: tunica albuginea thickness (TAT), seminiferous tubules diameter (STsD), Johnsen’s score, tubular differentiation index (TDI), spermiogenesis index (SPI), Sertoli cell index (SCI), meiotic index (MI), and mononuclear immune cells (MICs) in testes. The antioxidant status was examined by evaluating testicular levels of ferric reducing antioxidant power (FRAP) and catalase (CAT) activity.

**Results:**

Histological results of the testis from diabetic rats showed significant decrease in STsD, Johnsen’s score, TDI, SPI, SCI and MI, and significant increase in TAT and MICs, while administration of RJ significantly reverted these changes (p<0.05). RJ treatment markedly increased activity of CAT and FRAP. There were significant differences in FRAP levels among C (13.0±0.5), RJ (13.4±0.3), D (7.8±0.6) and D+R (12.4±0.7) groups (p<0.05).

**Conclusion:**

RJ improved diabetes-induced impairment in testis, probably through its antioxidant property.

## Introduction

Diabetes mellitus is a common health problem and a serious metabolic disorder associated with severe functional and structural complications (1). Diabetes mellitus has adverse effects on many organs, especially on the testis (2). Atalay Uslu *et al* reported that diabetes prevents spermatogenesis and decreases fertility rates by causing the thickening of basal membranes of seminiferous tubules and blood vessel walls, the formation of giant cells and severe degenerative injure in testicular tissue of rats (3).

On the other hand, it is well noted that there is a relationship between male infertility and diabetes mellitus in animal models (4). STZ-induced diabetes has also shown morphological testicular dysfunctions that practically affect all structures of the seminiferous tubules, including seminal linage and Sertoli cells (5).

Diabetes causes atrophy of sex organ, along with low levels of serum sex hormone such as testosterone, luteinizing hormone and follicle stimulating hormone in male rats (6, 7). Diabetes with various mechanisms caused testicular apoptosis and cell death. One of these mechanisms is that hyperglycemia can increase the production of ROS that cause cell apoptosis in different tissues including testis (8, 9). These disturbances in diabetic patient are related to early apoptosis (10).

During diabetes, chronic hyperglycemia causes an excessive production of free radicals, especially reactive oxygen species (ROS), for all tissues from glucose autoxidation and protein glycosylation (11). Damage of cellular organelles and enzymes and an increase in lipid peroxidation can be caused by high levels of free radicals and decreased efficiency of antioxidant enzyme defense (12).

During chronic diabetes, antioxidant defense system has a key role in the improving of long-term complications cause by ROS (13). The mammalian sperm cells contain a high amount of specific lipid composition which includes polyunsaturated fatty acid, plasmologen and sphingomyelin, but no antioxidant mechanism. Due to the high quantity of polyunsaturated fatty acid and low antioxidant capacity, mammalian spermatozoa are at a higher risk of peroxidative damage (14). A previous report indicated that high levels of ROS and free radicals have a destructive effect on sperm motility and decrease fertility in diabetic patients (15).

RJ is a honeybee product secreted from the hypopharyngeal and mandibular glands of the worker honeybees and serves as a primary food for the queen and larvae during their first three days of life (16). RJ has been recognized for having interesting pharmacological activities, such as anti-hypercholesterolemic, antioxidant and hypoglycemic properties (17-19). Moreover, this substance contains major proteins with high levels of peptides and essential amino acids, and high antioxidant properties and scavenging ability against free radicals (20). Polyphenols and phenols in major protein structures are responsible for antioxidant activity (21). Experimental studies showed that RJ exerts protective effects on different tissues, including anti-inflammatory effects and lowering of serum glucose levels (22, 23). There is no scientific data on RJ effect in treatment of diabetes-associated male reproductive dysfunctions such as histopathological changes in testis. 

Therefore, the present study aimed to assay the therapeutic potential of RJ on histopathological changes of rat testis with STZ-induced diabetes.

## Materials and methods

**Animals**

This experimental study was conducted with the approval of the local ethics committee on use and care for animal experiments at Kermanshah University of Medical Sciences. 

In total, 28 healthy adult male rats of the Wistar strain (190-210 gr) were used in this study. The animals were kept under standard conditions of 21±2^o^C temperature and 12 hr light/dark cycle with free access to commercial food and drinking tap water.

**Experimental design and diabetes induction**

Twenty eight male Wistar rats were randomly divided into four groups; (each group contain 8 rats): Control group (C): normal rats which were gavaged only 1ml distilled water for 6 wks. Royal jelly group (R): normal rats which were administered RJ at 100 mg/kg BW for 6 wks orally. Diabetic group (D): diabetic rats which were received 1 ml distilled water orally for 6 wks. RJ-treated diabetic group (R+D): diabetic rats which were given RJ (100 mg/kg BW) by gavage for 6 wks. 

For inducing diabetes, 14 rats were injected intraperitoneally with freshly prepared solution of streptozotocin (STZ; Sigma Chemical Company, St. Louis, MO, USA) at the a dose of 50 mg/kg BW in 5 mmol/L citrate buffer (pH=4.5) (24). After 72 hr of STZ injection, rats were fasted for 12 hr and the tail blood glucose levels were analyzed using a glucometer (IGM-0002A, GP5EAKFO9548). Rats with fasting blood glucose levels more than 250 mg/dl were considered to be diabetic.

Fresh RJ was obtained from a commercial firm in Iran, and stored at -4^o^C in a refrigerator until use. 100 mg/kg of RJ were dissolved in 1 CC distilled water and administered orally for 42 consecutive days to groups of RJ and RJ-treated diabetic rats (25). At the end of the experiment, all animals were anesthetized and their testes were dissected out by laporatomy. The right testis was collected for histopathological studies and the left testis was stored at -70^o^C until it was used for catalase (CAT) activity and ferric reducing antioxidant power (FRAP) analysis.

**H** **istopathological** **assay**

Testis was cleaned and fixed in 10% buffered formalin solution and were embedded in paraffin; then they were cut at 5 μm thickness; and stained with H&E (26). The diameters of fifty seminiferous tubules from each section were randomly examined by using an ocular light micrometer (27). The thicknesses of averagely fifty points of tunica albuginea from each section were measured by a micrometer. Mean of seminiferous tubules diameter (STsD) and mean of tunica albuginea thickness (TAT) were determined for each testis by Dino light software*.*

Thirty seminiferous tubules (STs) (×400) were randomly examined per section, their tubular differentiation index (TDI) and spermiogenesis index (SPI) were calculated as the percent number of STs that were showing more than three layers of differentiated germinal cells from spermatogonia type A (28, 29). To calculate the SPI, the ratio of the number of STs with spermatozoids to the empty STs were calculated (30).

Sertoli cell index (SCI) and meiotic index (MI) of 20 STs were randomly examined per section. SCI is the ratio of the number of germ cells to the number of Sertoli cells. To evaluate MI, the ratio of the number of round spermatids/primary spermatocytes was calculated (31). To obtain testicular damage and spermatogenesis, at least 70 STs were examined per section, and each sections were scored histopathologically using the Johnson score (32). Seminiferous tubules were scored on a scale of 1 to 10 according to Johnsen’s criteria; 1: no cells present; 2: Sertoli cells without germ cells present; 3: only spermatogonia present; 4: only a few spermatocytes present; 5: no spermatozoa or spermatids present but many spermatocytes; 6: only a few spermatids present; 7: no spermatozoa but many spermatids present; 8: only a few spermatozoa present; 9: many spermatozoa present but disorganized spermatogenesis; 10: full spermatogenesis and perfect tubules.

The numbers of mononuclear immune cells (MICs) per one mm^2^ of the interstitial connective testicular tissue were calculated by using morphometrical lens device at 100× magnification in at least 20 different fields, and the mean values were determined (33). Study of histopathological changes was carried out using the following parameters: shedding of immature germ cells inside the lumen of STs, seminiferous tubular atrophy, appearance of multinucleated cells, decline of germ cell and germ cell layers number.

**Assessment of catalase activity**

Testes tissue was sliced into pieces and homogenized in phosphate-buffered saline (0.1 mol/L, pH=7.4) in cold condition to give 10% homogenate (w/v). The homogenates were centrifuged at 10000 rpm for 10 min at -4^o^C and supernatant was processed for estimating CAT activity. The catalase (CAT) activity in homogenized testicular tissue was measured. The activity of CAT was based on the disappearance of hydrogen peroxide in the tissue homogenate (0.1 ml) of phosphate buffer (50 Mm; pH=7.0) and 2.9 ml H_2_O_2_ in phosphate buffer (30 mM; pH=7.0). The absorbance was read at 240 nm in a spectrophotometer (Pharmacia, Novaspec II, and Biochrom, England). Results were expressed as units/mg of tissue (33).

**Assessment of the ferric reducing antioxidant power**

The testicular tissue was weighed and homogenates (10 % w/v) were prepared using a glass homogenizer with ice-cold 1.15 % KCl. The FRAP level of testicular tissue was determined based on the change in absorbance at 593 nm due to the formation of a blue coloured Fe^2+^- tripyridyltriazine compound from colourless oxidized Fe^3+^ form through the activity of electron donating antioxidants.

Working FRAP reagent was prepared by mixing 25 ml acetate buffer (pH=3.6), 2.5 ml TPTZ (2,4,6-tripyridyl-s-triazine) in 2.5 ml ferric chloride (Fecl_3_) and 40 mM hydrochloride acid. Fifty μl of 10% homogenate was added to 1.5 ml freshly prepared reagent incubated at 37^o^C. After 10 min, the complex between Fe^2+^ and TPTZ gave a blue color. FeSO4 was used as a standard for FRAP assay and data were expressed as mM/gr of wet tissue. All solutions were used in the day of preparation (34).

**Statistical analysis**

All data are expressed as mean±SE and the difference for comparison was considered significant at p<0.05. For statistical analysis SPSS package (version 18) was used for one way analysis of variance (ANOVA) and Tukey test.

## Results

**Histopathological** **findings**

**Figure 1:**
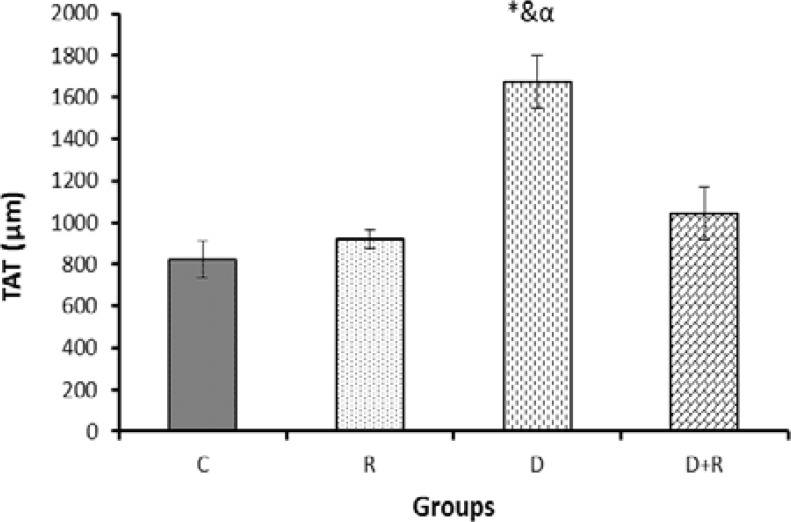
The thickness of tunica albuginea (TAT) measured by histological examinations of the testicular tissue in different groups.

**Figure 2:**
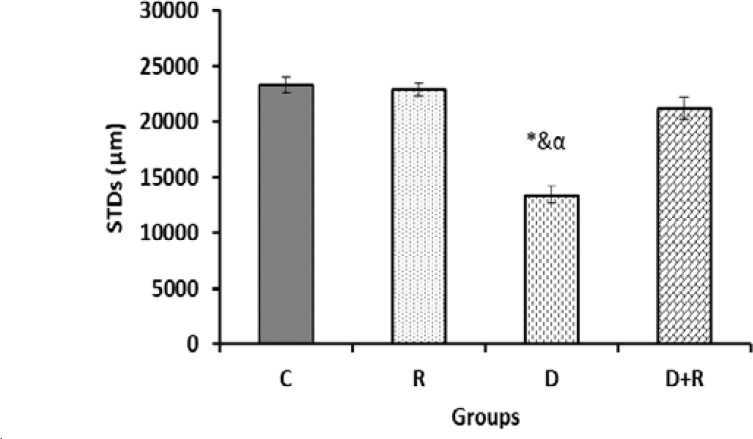
Data are presented as mean ±SE.

Histological examinations of the testicular tissue revealed that the thickness of tunica albuginea (TAT) was increased significantly in the group D. Treatment with RJ significantly decreased TAT compared to the diabetic rats (p=0.002) (Figure 1). In the group D, there was a significant decrease in the diameter of STs compared to the group C. The diabetic rats that received RJ showed a significant increase of the diameter of STs compared to diabetic rats (p=0.000) (Figure 2). 

The MI was significantly decreased in the group D compared to groups C and R. In the group D+R, the MI was increased in comparison with the group D. No significant difference was observed between this group and the C, R and D+R groups (p=0.02). The diabetic rats showed a significant decrease in SCI compared to the C and R groups. When the diabetic rats were treated with RJ for 42 days, the SCI increased compared to the diabetic rats (p=0.020).The mean value of SPI was significantly lower in the group D. However, RJ treatment significantly increased compared to the group D (p=0.001). The TDI in the group D was significantly decreased; whereas, it was increased in D+R group compared to the group D. There were no significant changes in group D+R in comparison with groups C and R (p=0.002). The mononuclear immune cells (MICs) count showed a significant increase in the group D (83.0±6.1 mm^2^) compared with C (14.3±1.5 mm^2^), R (13.0±1.1 mm^2^) and D+R (27.3±4.5 mm^2^) groups (p=0.000) (Table I).

**Figure 3:**
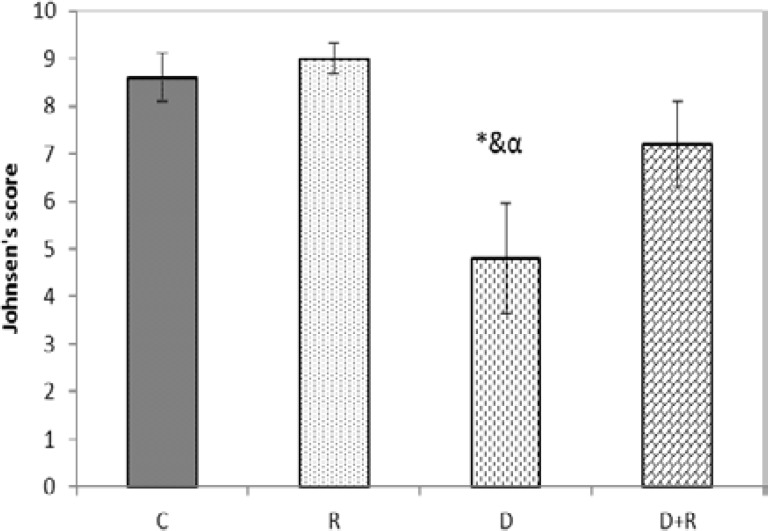
Effects of RJ on Johnsen's score in testis tissue of control and experimental groups of rats (N=7/group).

**Figure 4:**
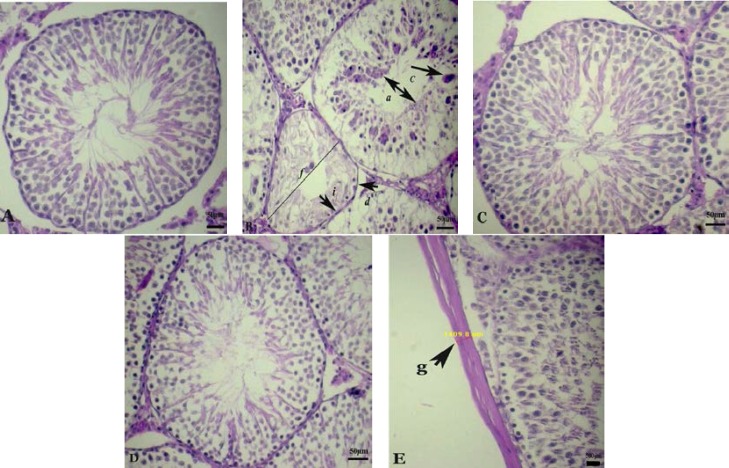
Photographs of several seminiferous tubules in rats stained with H&E (× 400). Testicular microscopic image of (A) normal rat testis of C group; (B); Diabetic rat testis with disorganization of the seminiferous tubule epithelium (a) and separation of the basement membrane (b), giant multinucleated cell in seminiferous tubule (c), seminiferous tubular atrophy (d), decline of germ cell layers number (h), spermatogenous arrest (i), the diameter of seminiferous tubules (f); (C) normal rat testis that received RJ; (D) diabetic rats treated with RJ showed improvement in seminiferous tubular structure; (E) the thickness of tunica albuginea indicated by arrows (g).

Mean Johnsen’s score of the group D was significantly lower as compared to the C, R and D+R groups. Mean Johnsen’s score of D+R group was closer to the Johnsen’s score of C group (p=0.007) (Figure 3). We observed the presence of degenerated epithelium (a) and separation of the basement membrane (b), appearance of giant multinucleated cells (c), seminiferous tubular atrophy (d), decline of germ cell layers number (h), spermatogenous arrest (i) and decrease the diameter of seminiferous tubules (f) in the diabetic rats compared to the control rats (Figure 4B). 

Another aspect of this study was the measurement of the thickness of tunica albuginea, which significantly decreased in diabetic rat testis (Figure 4E). Control and RJ group showed healthy histological structure of the seminiferous tubules and spermatogenesis (Figure 4A, 4C). The testicular sections of the RJ-treated diabetic rats showed remarkable improvement in STs structure as well as remarkable elevated in spermatogenic cell number (Figure 4D).

**Biochemical** **findings**

The CAT activity in the diabetic rats was significantly lower than other groups. Furthermore, the CAT activity was significantly increased in the diabetic rats treated with RJ compared to the diabetic rats. The CAT activity was significantly increased in RJ group compared to the control group (p=0.001). The results showed that the FRAP level of testis tissue was significantly decreased in the diabetic group as compared with control and RJ groups. The RJ increased the antioxidant power in RJ-treated diabetic group compared to the diabetic group (p=0.000) (Table II).

**Table I:** Effect of RJ on MI, SCI, SPI, TDI and MICs of seminiferous tubules in control group (C), royal jelly (R), diabetic (D) and royal jelly-treated diabetic (D+R) groups (n=7

**Groups **	**MI**	**SCI**	**SPI (%)**	**TDI (%)**	**MIC** **s (mm** ^2^ **)**
C	2.04 ± 0.02	12.01 ± 0.33	88.3 ± 4.5	90.33 ± 2.3	14.33 ± 1.45
R	2.09 ± 0.04	12.14 ± 0.42	89.5 ± 2.7	91.54 ± 3.9	13.00 ± 1.15
D	0.88 ± 0.17*&	8.75 ± 0.47*&	51.1±4.0*&β	52.20±4.1*&β	83.00 ± 6.08*&β
D+R	1.67 ± 0.42	10.92 ± 1.04	81.7 ± 4.8	76.50 ± 8.4	27.33 ± 4.48

Data are presented as mean±SE.

MI: Meiotic index

SCI: Sertoli cell index

SPI: Spermiogenesis index

TDI: Tubular differentiation index

MIC: Number of mononuclear immune cells.

* p<0.05 compared to C group.

&: compared to R group.

α : compared to D group.

β : compared to D+R group.

**Table II:** Effect of RJ administration on the FRAP levels and CAT activity in testicular tissue samples of control (C), royal jelly (R), diabetic (D), and royal jelly-treated diabetic (D+R) groups (n=7

**Groups **	**CAT** **(units/mg tissue)**	**FRAP (mM gr tissue)**
C	0. 61 ± 0.01	13.0 ± 0.5
R	0. 73 ± 0.02*β	13.4 ± 0.3
D	0. 32 ± 0.04*&β	7.8 ± 0.6 *&β
D+R	0. 57 ± 0. 03	12.4 ± 0.7

Data are presented as mean±SE.

CAT: Catalase

FRAP: Ferric reducing antioxidant power.

* p<0.05 compared to C group.

&: compared to R group.

α : compared to D group.

β : compared to D+R group

Data are presented as mean ±SE.

*p<0.05 compared to C group. &*:* compared to R group. α: compared to D group. β: compared to D+R group.

TAT: Tunica albugina thickness

C: Control group

R: Royal jelly groups

D: Diabetic group

D+R: Royal jelly-treated diabetic group.

*p<0.05 compared to C group. &*:* compared to R group. α: compared to D group. β: compared to D+R group.

STsD: Seminiferous tubules diameter.

C: Control group

R: Royal jelly groups

D: Diabetic group

D+R: Royal jelly-treated diabetic group.

Data are presented as mean±SE.

*p<0.05 compared to C group. &*:* compared to R group. α: compared to D group. β: compared to D+R group.

C: Control group

R: Royal jelly groups

D: Diabetic group

D+R: Royal jelly-treated diabetic group.

## Discussion

The present study was designed to determine the protective effects of RJ against testis damage in streptozotocin-induced diabetic rats. RJ increased STsD, Johnsen’s score, TDI, MI, SCI, and SPI and decreased TAT and MICs. In addition RJ elevated the CAT activity and ferric reduce antioxidant power level of testis tissue by decreasing the MDA level.

Previous studies have confirmed that STZ-induced diabetes mellitus changes in testis consist of decreased STsD, degeneration of germinal cells, interstitial edema and capillary congestion in rats (35). Moreover, diabetes effects on the testicular tissue have been attributed to insufficient production of insulin which in turn results in decreasing the endocrine function of both Leydig and Sertoli cells. Diabetes decreased serum levels of follicle-stimulating hormone (FSH), luteinizing hormone (LH) and testosterone and also FSH can severely affect spermatogenesis and the endocrine function of testis. Kühn-Velten showed that reduced insulin levels resulted in decreasing level of FSH in diabetic rats (7, 36).

Ricci *et al* reported that diabetes caused frequent abnormal histology and atrophy of the seminiferous tubules, decreased the tubules’ diameter and reduced spermatogenetic cell series (5). Diabetes mellitus caused a reduction of STsD and germinal epithelium height, cellular population of spermatogenesis, depletion of germ cells and interruption of spermatogenesis (37).

Evidence suggests that diabetes causes degeneration of germinal cells and formation of giant cells in testicular tissue (3). Our results clearly confirmed the decrease of disruption of spermatogenesis, STsD, TDI, SPI, SCI, MI, Jonson score, and formation of giant cells in STZ-induced diabetic rats. In addition, according to results of this study, diabetes results in an increase in TAT and MICs of the testis in diabetic rats. In the present study, we showed that RJ treatment markedly improved the above-noted parameters.

Reproductive system dysfunctions in diabetes have been reported as being associated with ROS. Oxidative stress is related to a rise in oxygen free radical production or an imbalance in the oxidant defense system (3). Studies have shown that activity of CAT have reduced in diabetic rats (33). The results of the present study showed a significant decrease in the CAT activity of the testicular tissue in diabetic group. Therefore, our findings supported the result of previous studies.

The RJ improved the testicular tissue damage induced by cisplatin, which is a chemotherapy drug, and increased CAT and glutathione peroxidase activities; pre-treatment with RJ, however, has shown to be more effective (25). El-Nekeety *et al* reported that RJ supplementation decreased MDA level and improved oxidative stress and antioxidant enzyme activities such as CAT, glutathione peroxidase (GPx) and superoxide dismutase (SOD) in fumonisin rat (38). Our findings revealed that RJ administration led to remarkable increase in CAT activity.

FRAP test has been used to assess the antioxidant power of the tissue (39). Previous studies have also indicated that the FRAP of plasma is significantly decreased in the diabetic rats as compared to control rats (40). In the present study, FRAP levels in the testicular tissue were significantly reduce in the diabetic group. However, it was observed that FRAP levels was increased in the rats treated with RJ.

In the testicular tissue, mean seminiferous tubule diameter and testicular biopsy scores are used to assess histopathological changes (32, 41). In the present study, mean Johnsen’s scores and STsD of RJ-treated diabetic rats were significantly higher when compared to diabetic rats. This study showed that RJ restored the diabetes-induced structural alterations in the testicular tissue. This effect could be attributed to the antioxidant properties and increasing activity of antioxidant defense systems of RJ.

## Conclusion

This research indicates that RJ administration attenuated diabetes-related testicular dysfunction and histopathologic changes. RJ renewed the activity of the CAT enzyme and significantly increased FRAP level in the testicular tissue of diabetic rats.
